# Giant localized electromagnetic field of highly doped silicon plasmonic nanoantennas

**DOI:** 10.1038/s41598-023-32808-w

**Published:** 2023-04-08

**Authors:** Ahmad E. Alsayed, AbdelRahman M. Ghanim, Ashraf Yahia, Mohamed A. Swillam

**Affiliations:** 1 Department of Physics, Faculty of Science, Ain Shams University, Cairo, 11566 Egypt; 2 Department of Physics, School of Sciences and Engineering, The American University in Cairo, New Cairo, 11835 Egypt

**Keywords:** Nanoscience and technology, Optics and photonics

## Abstract

In this work, we present the analysis and design of an efficient nanoantenna sensor based on localized surface plasmon resonance (LSPR). A high refractive index dielectric nanostructure can exhibit strong radiation resonances with high electric field enhancement inside the gap. The use of silicon instead of metals as the material of choice in the design of such nanoantennas is advantageous since it allows the integration of nanoantenna-based structures into integrated-optoelectronics circuits manufactured using common fabrication methods in the electronic industry. It also allows the suggested devices to be mass-produced at a low cost. The proposed nanoantenna consists of a highly doped silicon nanorod and is placed on a dielectric substrate. Different shapes and different concentrations of doping for the nanoantenna structures that are resonant in the mid-infrared region are investigated and numerically analyzed. The wavelength of the enhancement peak as well as the enhancement level itself vary as the surrounding material changes. As a result, sensors may be designed to detect molecules via their characteristic vibrational transitions. The 3D FDTD approach via Lumerical software is used to obtain the numerical results. The suggested nanoantennas exhibit ultra-high local field enhancement inside the gap of the dipole structure.

## Introduction

The optical properties of metallic nanoparticles have been widely investigated in recent years^1^. The development of metamaterials research has resulted in an understanding of various phenomena that are not attainable with natural materials. With recent technological advances, it is now possible to establish nanodevices and subwavelength structures with adequate precision. These nanostructures can include nanoantennas, plasmonic devices, metamaterials, and terahertz filters^2,3^. Plasmonic devices direct and control light at a metal/dielectric interface. Plasmonic nanostructures have been employed in a variety of applications, including power splitters^4^, 3D chips^5^, and solar cells^6^. Plasmonic structures efficiently promote interactions between nanodevices and the propagating radiation by compressing light into subwavelength volumes^7,8^.

Metals' plasmonic characteristics have drawn a lot of interest recently because of their ability to create localized surface plasmon resonance (LSPR). To control LSPR intensity and confine it, nanoantennas will be a good choice due to their light-directing and harvesting properties, therefore they are involved in a variety of applications, including trapping light, near-field manipulation, and nanoscale sensing^9^. Due to weak compatibility with semiconductor manufacturing techniques and significant dissipation at optical and infrared wavelengths, the usage of metals like gold or silver is still ineffective^10^. So, the research is oriented to study LSPR in highly doped semiconductors, especially silicon. The losses of the dielectric constant in the visible and infrared regions are low compared to the plasmonic metals^1^. The LSPR resonance is affected by some parameters like the constituent substance, the shape of the nanoantenna structure and its size, and the concentration of doping carriers. The highly doped silicon is tunable in the infrared range. According to the Drude model the LSPR region resonance change by altering the doping whereas the dopant concentration between ${10}^{18}$ to ${10}^{19}cm^{-3}$ the LSPR resonance will be in far-field infrared and by increasing the concentration of the doping to ${10}^{20}{\mathrm{ cm}}^{-3}$ the resonance range varying to the mid-infrared range^11^.

Gas sensing is found essential in the identification of hazardous gases in the environment as well as medical conditions^12^. Some of the most hazardous greenhouse gases, which endanger environmental stability, can be detected using gas sensors. Metal-oxide, capacitance, and electrochemical-based gas sensors are among the several gas sensing techniques. Among these sensing methods, the optical approach is particularly significant since it is a rapid, dependable, and extremely sensitive way of sensing. The optical gas sensor, on the other hand, is typically utilized for sensing several greenhouse gases, such as ${\mathrm{CO}}_{2}, {\mathrm{CH}}_{4},\mathrm{ and }{\mathrm{H}}_{2}\mathrm{S}$. These gases often absorb in the infrared (IR) or ultra-violet wavelength ranges^11^.

In this study, We will not condone the dielectric environment that also affects the LSPR frequency which is an essential parameter for gas sensors. A gas sensor is built to sense the different gases and select their concentration using dielectric/metallic or highly doped semiconductor nanoantennas, the first resonant in the visible region while the other in the infrared region. In addition to enhanced electric fields, a shorter resonant linewidth is desirable for specific applications. In gas sensing applications, for example, the detection length is defined not only by the LSPR's sensitivity to the local dielectric material but also by the resonant linewidth^13^. A narrower linewidth can detect smaller shifts^12^.

Doped Silicon was used to build nanoantennas that support plasmonic phenomena reported in metallic nanostructures and achieve comparable enhancement shifted to the mid-IR spectral region rather than the visible range^14^. The resonance frequency of the highly doped silicon nanoantenna fluctuates as the refractive index of the environment surrounding it changes. We suggest that the gas sensors based on highly doped semiconductors are better than ones based on plasmonic materials because if we can control the concentration of the doping, we can also governate the resonance region^10,11,15^. So, we rely on the concertation of $5\times {10}^{20}$ ${\mathrm{cm}}^{-3}$ which affects the mid-infrared (MIR). In MIR each gas owns a unique fingerprint.

## Numerical method

**Figure 1:**
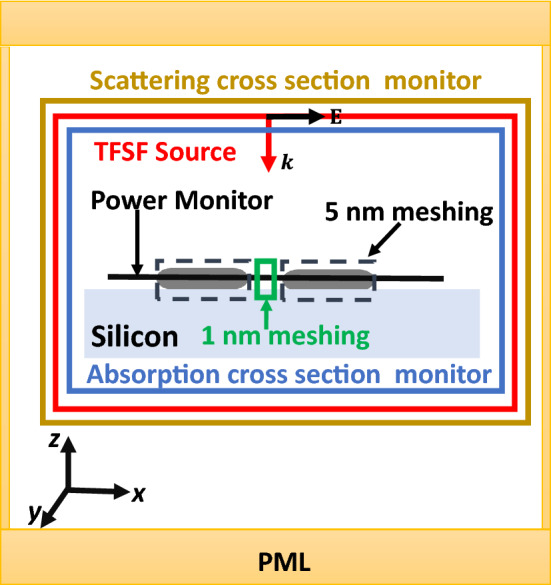
The FDTD Lumerical simulation setup is depicted schematically. Total-field scattered-field source (TFSF) is applied. To model a relatively large substrate, silicon extends beyond the simulation region and PML boundaries.

To study the optical properties and the near-field calculations of the nanoantenna, three-dimensional finite-difference time-domain simulations were conducted using a commercially available Maxwell equations software program (Lumerical FDTD Solutions)^16^. We first investigate the optical properties of the dipole antenna with a total field scattered field (TFSF) source polarized along the *z*-direction, as illustrated in Fig. 1. The incident radiation spectrum covers the wavelength range 1 μm to 15 μm. The 3D simulation box is bounded by anti-symmetric, symmetric, and perfect-matched boundary conditions (BCs) in *x*, *y*, and *z* directions, respectively in order to minimize the computation time. Mesh override sections covering distinct volumes of the structure were employed to achieve fine resolution. A mesh size of 5 nm was employed around each pole of the nanoantenna and 1 nm inside the gap. The near-field (wavelength-dependent intensity enhancement) inside the gap is obtained by the field and power monitor.

The absorption cross-section of a structure is calculated by multiplying the power passing through the silicon-based plasmonic nanoantenna structure by the intensity of the plane wave. In the simulations, we insert a power monitor box around the nanoantenna (6 two-dimensional monitors altogether) and then estimate the transmission of each monitor. The software's transmission radiation returns the amount of power transmitted via a power monitor or a profiled monitor, normalized to the source power. The absorption cross-section can be calculated by adding the six transmissions and multiplying by the source area. The scattering cross-section data could be produced similarly, with the main difference being that the monitors are located only in the scattered light zone. The doped silicon antenna and the dielectric substrate are now both covered by the matching scattering power monitor box^17^.

**Figure 2:**
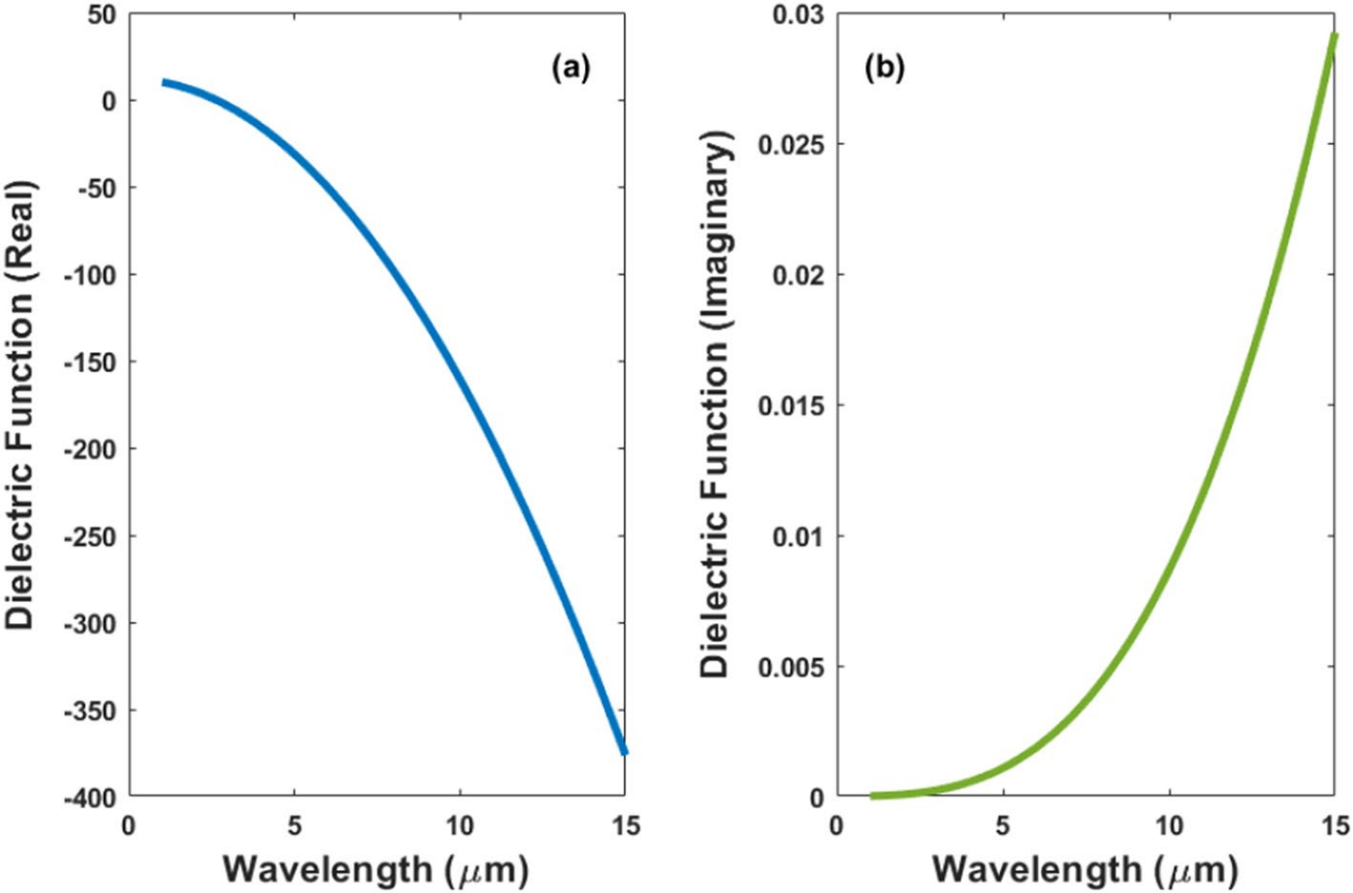
Material dispersion of highly doped silicon with a concentration of $5\times {10}^{20}$ ${\mathrm{cm}}^{-3}$. For wavelengths higher than 3 μm, the real part of the dielectric function becomes negative.

In this paper, we are dealing in general with dipole antenna with different shapes created with highly doped silicon placed on different substrates. Figure 1 illustrates a schematic diagram of a highly doped silicon nanorod antenna on top of a silicon substrate. The Palik model^18^ was used to calculate the real and imaginary refractive indices of the silicon (Si) substrate and the free space is supposed to be the surrounding medium with *n* = 1. We used the most realistic high-doping concentration to achieve plasmonic frequency with the shortest wavelength in the MIR range. Figure 2 depicts the material dispersion of phosphorus-highly doped silicon with a concentration of $5\times {10}^{+20} {\mathrm{cm}}^{-3}$ as defined using the Drude model for permittivity^11,19^:

1 $$\varepsilon \left(\omega \right)={\varepsilon }_{s}\left(1-\frac{{\omega }_{P}^{2}}{{\omega }^{2}+i\omega\Gamma }\right), {\omega }_{P}^{2}=\frac{n{e}^{2}}{{\varepsilon }_{s}{\varepsilon }_{0}{m}^{*}(n)}$$

2 $$\varepsilon \left(\omega \right)={\varepsilon }^{\mathrm{^{\prime}}}+i{\varepsilon }^{\mathrm{^{\prime}}\mathrm{^{\prime}}}={\varepsilon }_{s}\left(1-\frac{{\omega }_{P}^{2}}{{\omega }^{2}+{\Gamma }^{2}}\right)+i{\varepsilon }_{s}\left(\frac{\frac{\Gamma {\omega }_{P}^{2}}{\omega }}{{\omega }^{2}+{\Gamma }^{2}}\right)$$

where ${\varepsilon }_{\infty }=11.7$ F/m is the dielectric permittivity at high frequency, ${\varepsilon }_{\mathrm{s}}$ is the background dielectric constant, *N*_*d*_ represents the free carrier concentration, ${\omega }_{p}$ is the plasma frequency, ${\varepsilon }_{0}$ represents free space, and ${m}^{*}$ denotes the electron effective mass; $\Gamma$ is the collision frequency in rad/s and is defined as $\Gamma =q/({m}^{*}\mu )$ where $\mu$ represents carrier mobility and q denotes electron charge. In the case of a doping concentration (*N*_*d*_) $5\times {10}^{20}$ ${cm}^{-3}$, ${\omega }_{p}=2.474\times {10}^{15}$ rad/s and $\Gamma =9.456\times {10}^{9}$ rad/s. It can be shown from Fig. 2 that, for wavelengths higher than 3 μm, the highly doped silicon has a large negative real and a small positive imaginary dielectric function that can support plasmon excitation, which is a collective excitation of conduction electrons^20^.

Doped silicon performs better than metals in plasmonic-based gas sensor applications at mid-infrared^11,15^. In general, semiconductors do not have rough surfaces, which reduce scattering losses as compared to noble metals. Furthermore, silicon offers various benefits, such as CMOS compatibility and easy fabrication using standard Si fabrication techniques. Furthermore, working in the mid-IR region while utilizing doped silicon material allows for the development of integrated plasmonic devices on the microscale. This combination facilitates the fabrication of many conventional plasmonic devices^15^.

The suggested doped silicon nanorod antenna can be fabricated using electron-beam lithography (EBL)^10,21^. Lithography is one of the most straightforward methods for fabricating nanostructures since it provides excellent repeatability as well as the capacity to create nanostructures of complex forms using a combination of lithographic techniques. Traditional lithographic methods have been used effectively to create single nanoparticles of various shapes. For example, silicon nanorod antennas with an outside diameter of 30 nm and a gap between the nanorods > 20 nm were created using a combination of electron-beam lithography and reactive-ion etching^22^. Another suggested fabricated method is the direct laser ablation which was used in the first experiments to fabricate high-index nanoparticles: an ultrashort-laser-pulse-induced material fragmentation into spherical nanoparticles and their deposition near the focal region^10,23^. This experiment demonstrated the use of laser ablation in the synthesis of high-index nanoparticles with the optical response (scattering efficiencies, *Q* factors, and so on) in the visible and infrared spectral ranges. Another approach that can be used to produce high-index nanoparticles on a large scale is dewetting of a thin layer. This mechanism indicates nanoparticle agglomeration during thin film heating due to the minimization of the total energy of thin film surfaces, including a film-substrate interface^24,25^.

## Results and discussions

**Figure 3:**
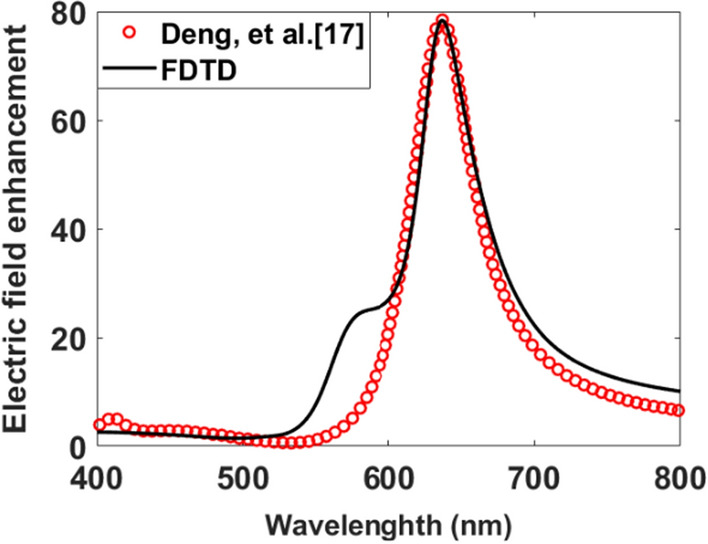
Variation of the wavelength-dependent intensity enhancement of plasmonic nanoantenna-dielectric nanocavity^17^ and by using the FDTD method.

In order to verify the simulation results calculated by the 3-D FDTD method^26^, plasmonic nanoantenna-dielectric nanocavity designed by Yan-Hui Deng, et al.^17^ is initially considered. The dielectric substrate disc has a diameter and height of 160 nm. The plasmonic nanoantenna has been designed using two identical Au nanorods. Each Au nanorod has a total length and diameter of 26 nm and 10 nm, respectively. The hemisphere at both ends of the nanorod has a diameter of 10 nm. As illustrated in Fig. 3, the gap distance between the two nanorods is 5 nm. A spacer is placed between the dielectric nanodisk and the plasmonic nanoantenna. The spacer's height is 5 nm with a refractive index of *n* = 1.46. The dielectric nanodisk has a refractive index that is assumed to be *n* = 3.3^17^. The wavelength-dependent intensity enhancement computed by the FDTD approach and by Yan-Hui Deng, et al.^17^ is shown in Fig. 3. It can be noted that a good agreement can be observed between our results and those published by^17^, indicating that our model is accurate.

To first investigate the optical properties and the local field enhancement, the dipole nanoantenna is illuminated by (TFSF) source incident normally in the *z*-direction and is modeled using the 3-D FDTD approach via the Lumerical package as shown in Fig. 1^16^. In this paper, the dipole nanorod antenna with highly doped silicon material placed on a silicon substrate is introduced as an alternative solution to enhance the near-field intensity with low loss in the MIR range. The geometrical parameters of the dipole nanorod design are as follows: the substrate dimensions are 2000 nm $\times$ 1700 nm $\times$ 90 nm in *x*, *y*, and *z* directions, respectively and it is modeled by Palik^18^. The nanoantenna is structured by two identical highly doped silicon nanorods. Each nanorod is *L* = 222.5 nm long and has a radius of 15 nm, which is the same as the radius at the two ends. As seen in Fig. 1, the surface space between the two nanorods is *g* = 30 nm.

**Figure 4:**
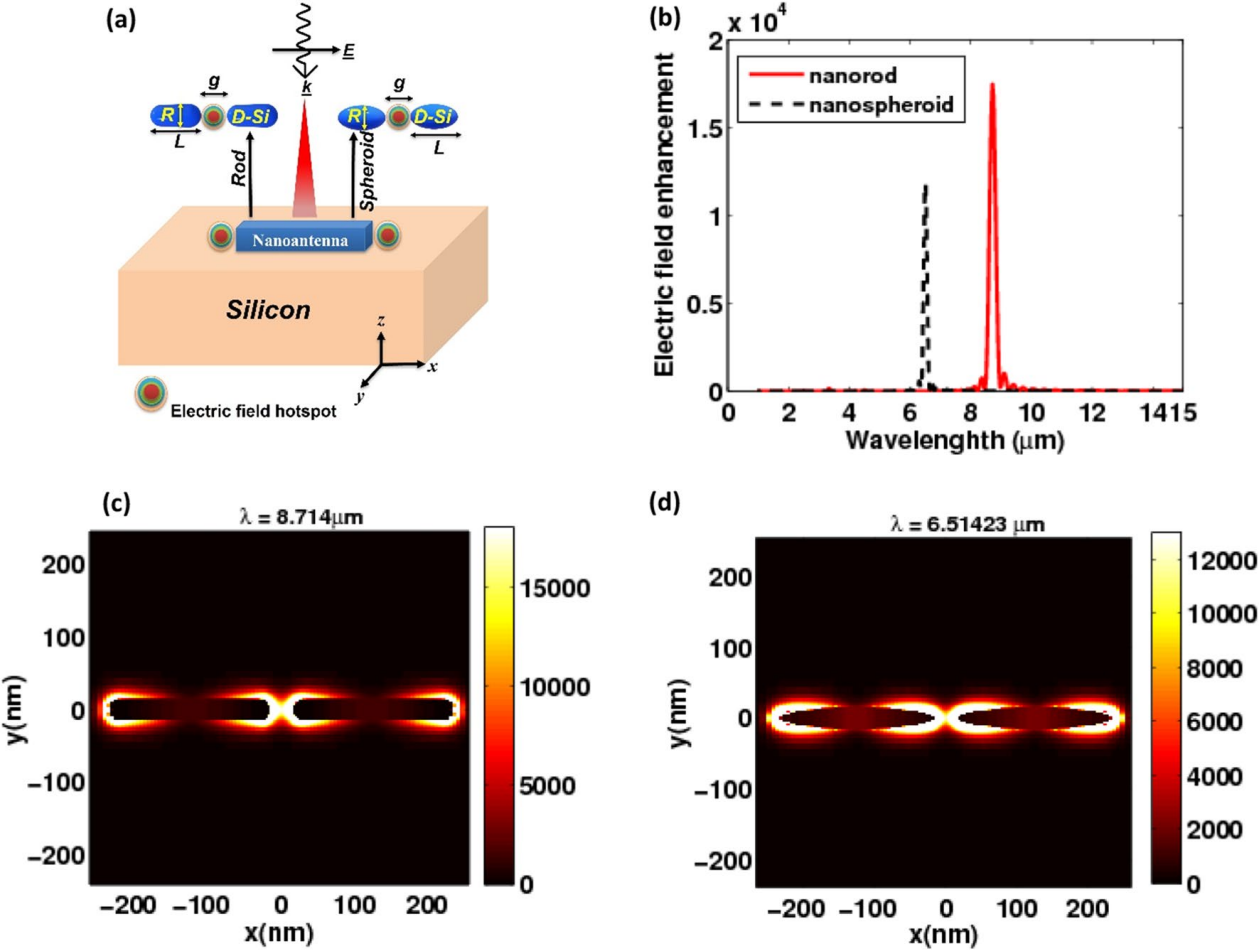
(**a**) Schematic of a nanoantenna structure consisting of dielectric nanorod or dielectric nanospheroid. (**b**) a comparison between the electric field enhancement at the center of the gap as a function of the wavelength for the nanorod and the nanospheroid. (**c**) the profile of the local field enhancement inside the gap of the nanorod. and (**d**) the profile of the local field enhancement inside the gap of the nanospheroid.

The local field intensity enhancement at the center of the gap relative to the incident field at the MIR of the nanorod, as well as the field profile at the resonance wavelength, are plotted in Fig. 4. We first start our investigation with a nanorod on a silicon (Palik) substrate which shows a narrow peak resonant at 8.71404 μm as shown in Fig. 4b. The electric near-field intensity is confined in the center of the gap between nanorods which shows a high enhancement of about 17,464.5 times compared to the normalized electric field (Fig. 4b). This strong near-field in the gap between nanorods indicates a localized surface plasmonic resonance (LSPR). The simulation profile demonstrates that at MIR wavelengths, the system exhibits the advantages of a coupled dipolar resonance, as shown in Fig. 4c. Due to the coupling between the two adjacent doped silicon nanorods, a plasmonic mode at a MIR wavelength is created^(9)^. Additionally, As the direction of the incident electric field (polarization) is parallel to the long axis of the nanorod antenna, free electrons accumulate along the shorter edge (width), stimulating the longitudinal plasmon mode at longer wavelengths (Fig. 4c)^9^. In general, this results in substantial near-field enhancement, which is useful in sensitivity applications.

We also highlight how the shape and geometry of the nanoantenna influence the resonance properties^27^. If we adjusted the length, gap, and substrate of the previous nanoantenna geometry, the resonance wavelength obtained is blue-shifted to 6.51423 µm in the case of spheroidal shape (see Fig. 4b). The near field was almost distributed equally on the two edges of each nanospheroid, as shown in Fig. 4d. The local field enhancement inside the gap center for the nanospheroid is investigated and analyzed. It is found from Fig. 4b that the maximum normalized field intensity is dropped to 11,682.6 compared to the nanorod shape.

**Figure 5:**
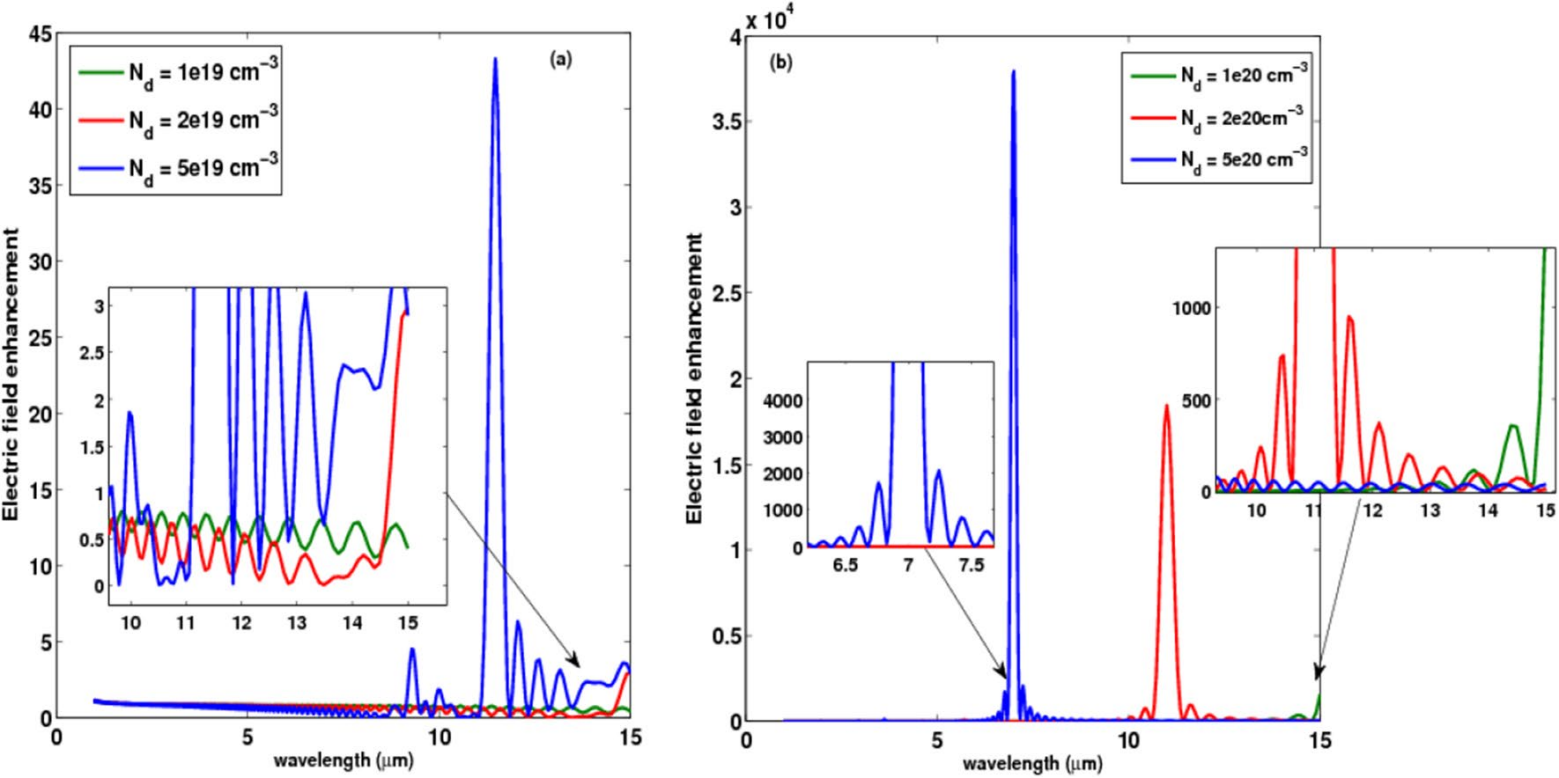
Electric field enhancement at the center of the gap (*x* = 0, *y* = 0) versus wavelength for different doping concentrations. The polarization of the incident plane wave is along the antenna (*x-*axis).

The nanorod antenna design on a hybrid substrate with varying doping concentrations is also modeled in our computation, including $1\times {10}^{19}$ ${\mathrm{cm}}^{-3}$, $2\times {10}^{19}$ ${\mathrm{cm}}^{-3}$, $5\times {10}^{19}$ ${\mathrm{cm}}^{-3}$, $1\times {10}^{20}$ ${\mathrm{cm}}^{-3}$, $2\times {10}^{20}$ ${\mathrm{cm}}^{-3}$, and $5\times {10}^{20}$ ${\mathrm{cm}}^{-3}$. Figure 5 depicts the field intensity at the center of the gap as a function of doping concentration. The results indicate that as the doping concentration increases, the resonance wavelength is a blue shift. The increase in doping concentration corresponds to an increase in field intensity enhancement, and the best concentration of doping obtained is $5\times {10}^{20}$ ${\mathrm{cm}}^{-3}$, as shown in Fig. 5, which is employed in all nanoantenna structures.

The intensity ripples shown in Fig. 5 are attributed to the surface plasmons (SPs) which are the collective oscillations of free electrons evanescently coupled to a metal surface that interact with the electromagnetic field to create localized surface plasmon resonances (LSPRs)^28^. Collective oscillations of conduction band electrons are confined to the nanoscale structure boundaries, resulting in intense electromagnetic fields. Similarly, localized magnetic fields can be generated by induced electric oscillation currents in plasmonic nanostructures. Localized Surface Plasmons (LSPs) are standing wave Surface Plasmon Polariton (SPP) modes at confined nanostructure boundaries. As a result, the same nanoparticles can manage a wide range of LSP modes with varying orders of the standing wave at different optical wavelengths as shown in Fig. 5^29^.

**Figure 6:**
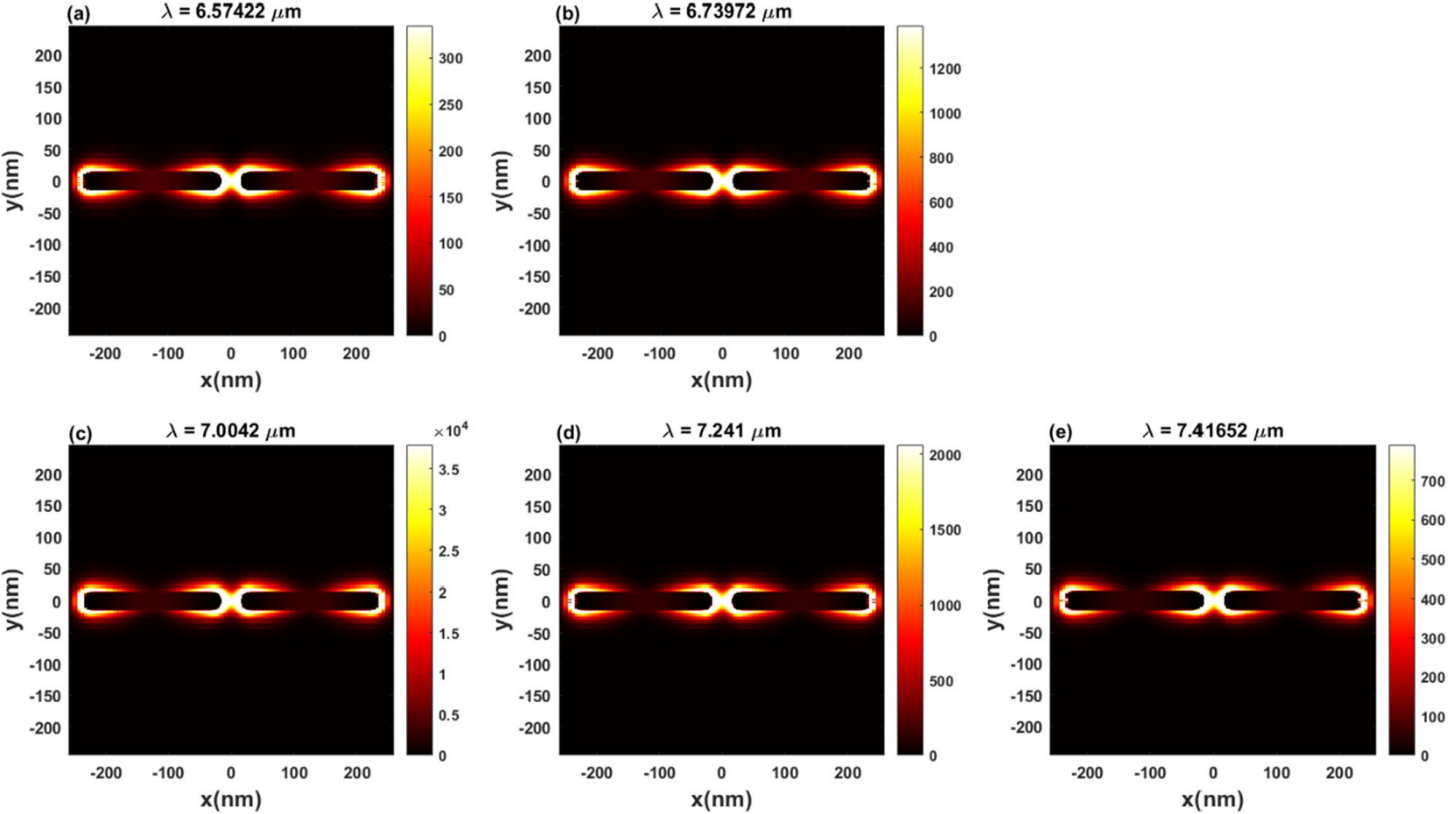
The distribution of the local electric field in the *XZ*-plane induced by normal incidence at the resonant wavelengths.

The field enhancement of the rod with concentration $5\times {10}^{20} {\mathrm{cm}}^{-3}$ on a hybrid substrate shown in Fig. 5 exhibits a mean peak resonance and multiple plasmon modes at different wavelengths.  The field distribution in Fig. 6a,b are related to the two peaks at $\lambda = 6.57422 \mathrm{\mu m}$ and $\lambda = 6.73972 \mathrm{\mu m}$ before the major mode at  $\lambda = 7.004 \mathrm{\mu m}$ and its profile field is shown in Fig. 6c. Figure 6d,e illustrate the field distribution of the peaks at $\lambda = 7.241 \mathrm{\mu m}$ and $\lambda = 7.41652 \mathrm{\mu m}$.

**Figure 7:**
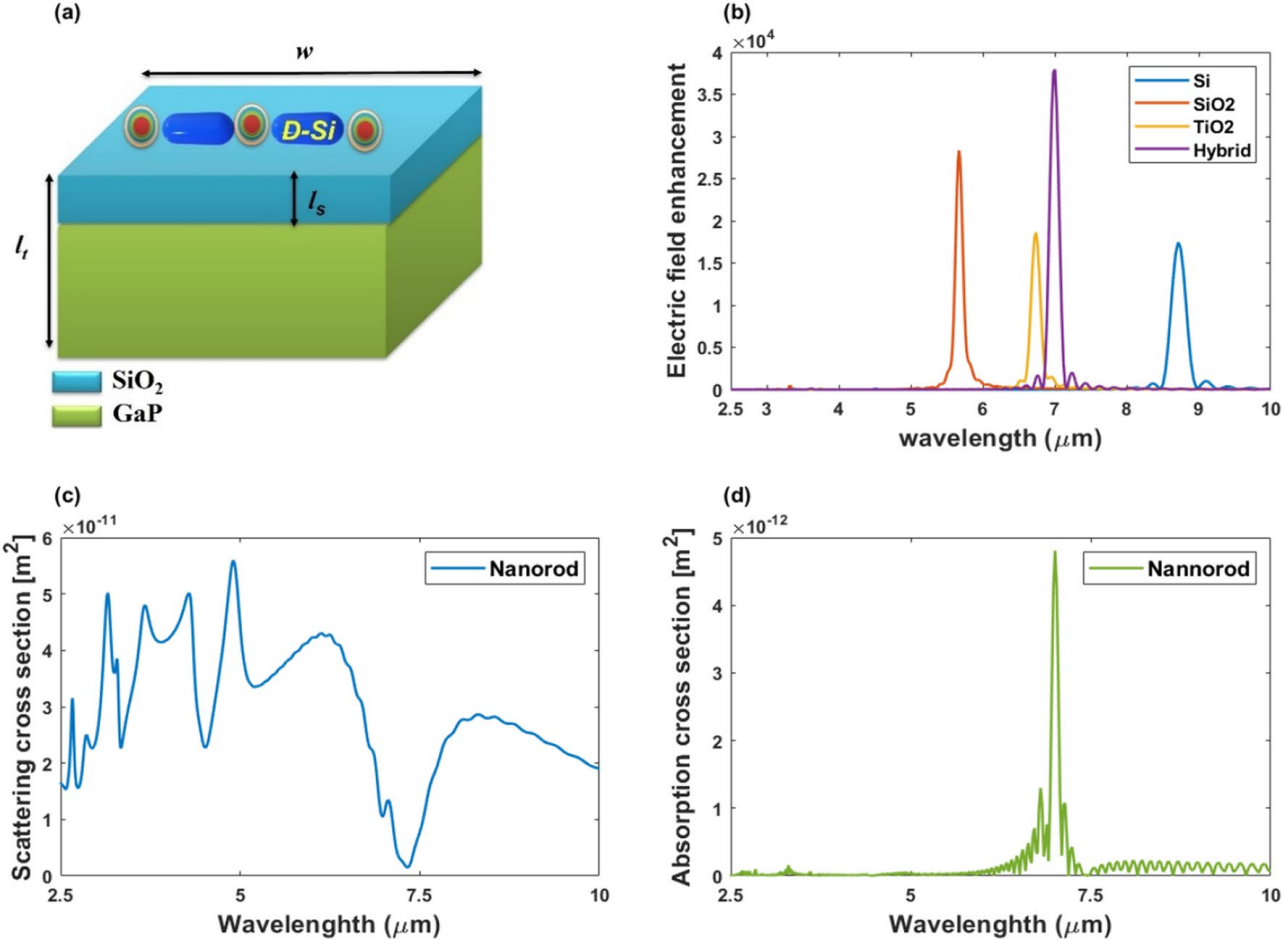
(**a**) Schematic of the nanorod antenna on the top of the hybrid substrate, (**b**) variation of the wavelength-dependent intensity enhancement with different substrate materials. (**c**) The scattering cross-section of the nanorod on the hybrid substrate as a function of the wavelength. (**d**) The absorption cross-section of the nanorod on the hybrid substrate as a function of the wavelength.

In addition to the variation of the nanoantenna design, the variation of the surrounding substrate has been reported. The surface plasmon resonance of the coupled nanoparticle pair was found to vary depending on the substrate material. It is more sensitive to its surroundings/substrate than an isolated nanoparticle. As a result, the influence of the substrate material on field enhancement inside the gap center is also studied. The same nanoantenna as shown in Fig. 4a is placed on the same substrate dimensions, however with different materials: Si, SiO_2_, TiO_2_, and hybrid substrate as recommended in^17^. The hybrid substrate is composed of two materials; a substrate with a refractive index of *n* = 3.3 and a very thin spacer with a refractive index of *n* = 1.46^17^. The substrate (*n* = 3.3) and spacer (*n* = 1.46) are about *l*_*t*_ = 980 nm and *l*_*s*_ = 5 nm in height and both widths are *w* = 2000 nm, as shown in Fig. 7a. Figure 7b exhibits the light intensity inside the gap center as influenced by various substrates. As demonstrated in Fig. 7b, the hybrid substrate exceeded the silicon substrate, doubling the enhancement in the field, with the intensity peak reaching 37,930.1 and being resonated at approximately 7.0042 µm. The study found that substrate materials such as SiO_2_, and TiO_2_ exhibit significant enhancement behaviors in our investigated frequency range.

A normal incident TFSF excites the nanoantenna. In order to have a better understanding of the multipole LSPRs in the nanoantenna presented in this research work, The scattering and absorption cross-section spectrums of the nanorod antenna on the hybrid substrate are estimated as can be seen in Fig. 7c,d. The scattering cross-section in Fig. 7c depicts the resonance positions under normal excitation. The scattering spectrum shows different resonances with wavelengths of 3.16, 3.68, 4.28, 4.9, 6.12, and 8.3 µm, corresponding to the multipolar modes^29^. Figure 7d depicts the absorption spectrum of the coupled system. The spectra show a sharp peak at *λ* = 7.004 µm.

**Figure 8:**
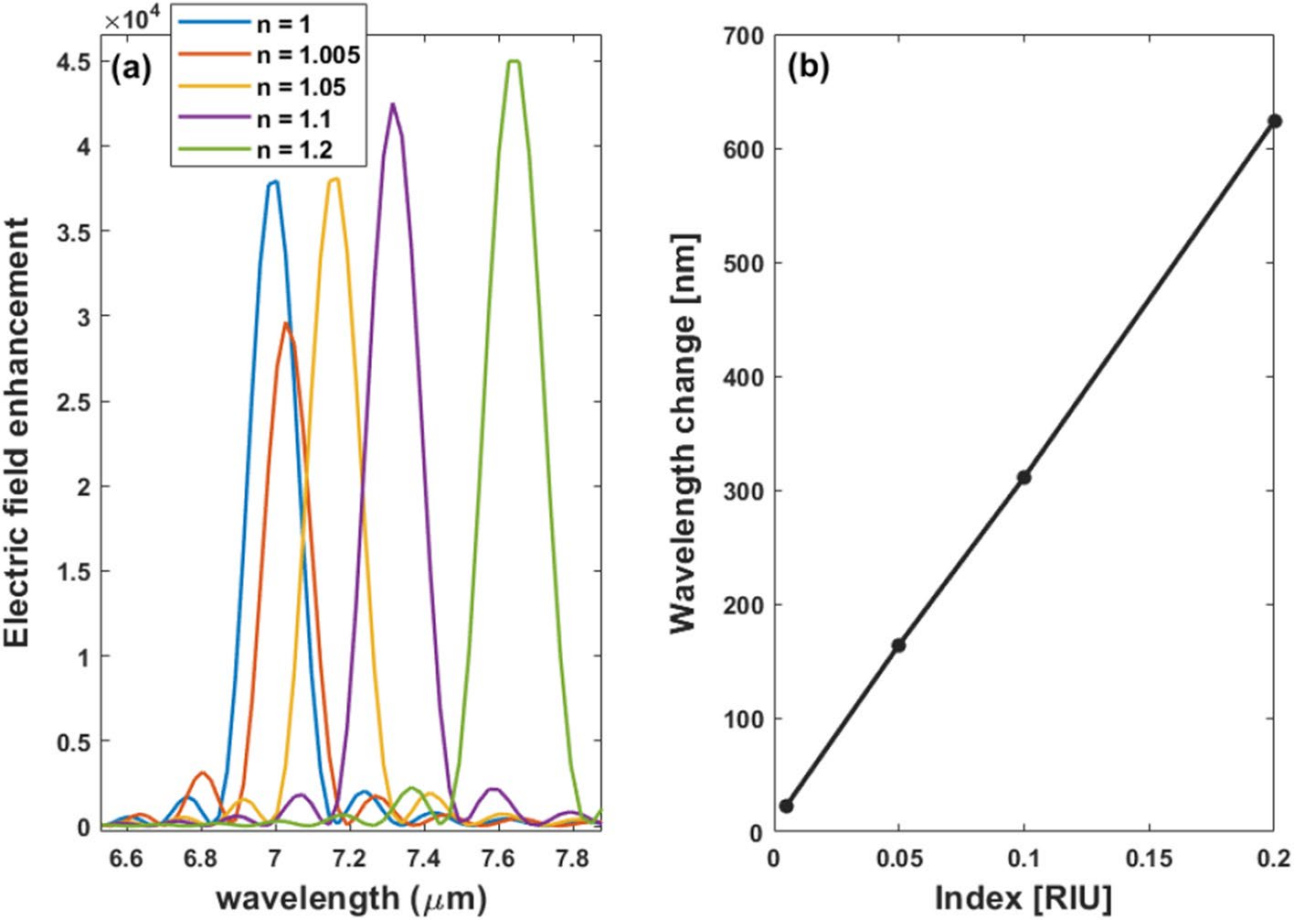
Electric field enhancement at the center of the gap (*x* = 0, *y* = 0) versus wavelength for different medium refractive indices and (**b**) shows the resonance wavelength change with varying RI for the nanorod on the hybrid substrate.

It can be shown from Fig. 7b that, the field exhibits significant confinement at the resonance wavelength of 7.004 μm. This indicates that the mentioned design will be sensitive to any slight change in the surrounding refractive index (RI). We studied the effect of intensity for the nanoantenna by changing the refractive index of the surroundings using FDTD simulations. Figure 8a reveals the difference between air and gas field intensities as a function of the wavelength, and as noticed, the resonance wavelength exhibits a red shift due to changes in the RI of the surroundings related to an increase in the electric field enhancement. In order to get the sensitivity of the nanorod in accordance with the change in RI, the shift in the resonance wavelength is divided by the refractive index change (*S* = *Δλ*/*Δn* nm/RIU). As can be shown in Fig. 8b, the variations in resonant wavelength are fitted linearly as a function of medium refractive index, revealing a high refractive index sensitivity of 3118 nm/RIU.

**Figure 9:**
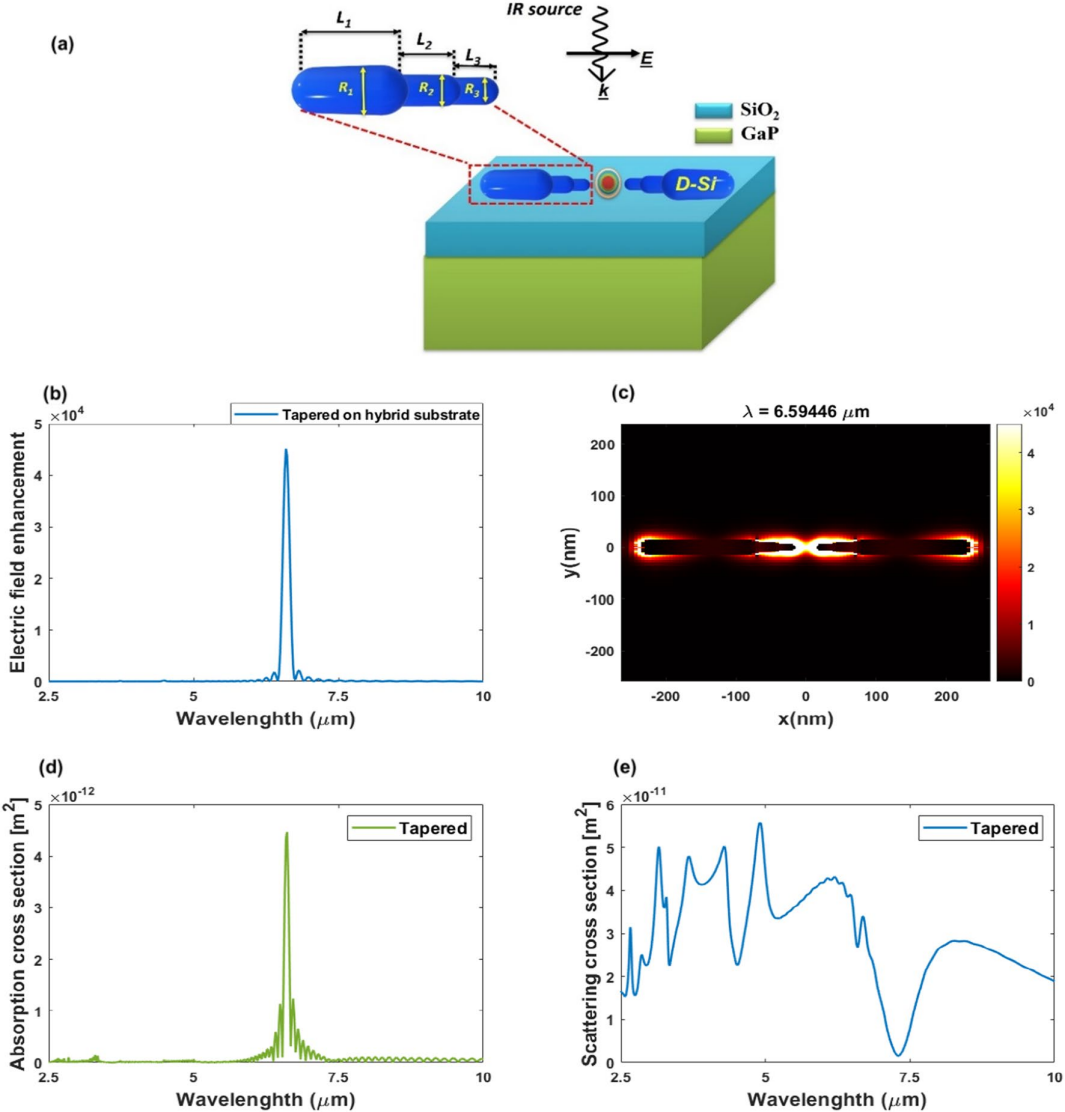
(**a**) The proposed tapered nanorod antenna on hybrid substrate, (**b**) electric field enhancement inside the gap center as a function of the wavelength, and (**c**) the field profile of the tapered nanoantenna at the resonance wavelength. (**d**) The scattering cross section of the tapered nanorod antenna on hybrid substrate as a function of the wavelength. (**e**) The absorption cross section of the tapered nanorod antenna on hybrid substrate as a function of the wavelength.

By adding more steps with smaller dimensions to the standard dipole design, a highly confined field is obtained inside the center of the gap of the suggested nanorod antenna design (tapered nanorods) after the simulations. This improvement is the result of the surface plasmon that the plane source stimulated. Accordingly, the field values are recorded across the specified frequency range between the two nanorods at the previously indicated spacing gap. The three tips that form a tapered nanorod have lengths of *L*_1_ = 172.5 nm, *L*_2_ = 30 nm, and *L*_3_ = 20 nm and have radii of *R*_1_ = 15, *R*_2_ = 10, and *R*_3_ = 7.5 nm. The proposed tapered-dipole nanoantenna, which is regarded as a modified form of the conventional dipole with two additional steps of smaller dimensions introduced, is shown in Fig. 9a along with its design characteristics^30^.

The near-field intensity enhancement of the tapered-dipole nanoantenna is investigated in this study over the MIR wavelength range from 1 to 15 µm. The tapered dipole nanoantenna is optimized to achieve maximum near-field intensity over the MIR spectrum. Figure 9 depicts the field intensity spectra of the tapered nanoantenna installed on top of the hybrid substrate. It is evident from Fig. 9b that an intensity peak appears at the resonance wavelength of 6.59446 µm, which corresponds to the dipolar mode. The near-field intensity at resonance wavelength is around 45,000, which is more than the previously reported nanorod antenna in the MIR band shown in Fig. 7. As indicated in the electromagnetic theory, this field enhancement can be attributed to the field accumulated at the tips of the doped silicon nanorods^31^. Based on this phenomenon, the suggested design was developed by incorporating additional steps of smaller dimensions into the conventional dipole design, which results in a large divergence of surface current over the nanoantenna surface due to the multiple thickness grading involved in the design^30^. The electric field profile for tapered-dipole nanoantennas at the resonance wavelength is shown in Fig. 9c. The electric field is accumulated with high intensity around the three tips of the nanoantenna, as seen in this figure, due to a high divergence of surface current^30^.

For the tapered nanoantennas, the scattering and absorption cross-sections are estimated versus the wavelength. Figure 9d,e depict the results. There are multi-resonance modes shown in the tapered structure with wavelengths of 3.155, 3.67, 4.28, 4.9, 6.1, and 8.3 µm. Figure 9e shows the coupled system's absorption spectrum. A strong peak can be seen in the spectra at *λ* = 6.59446 µm.

**Figure 10:**
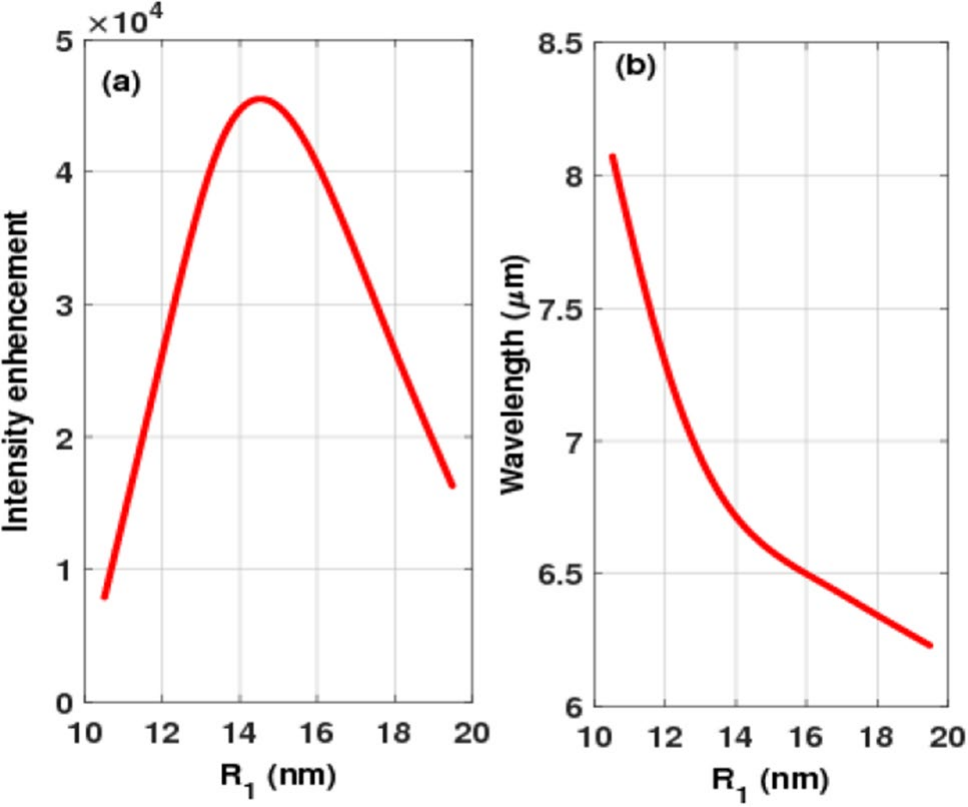
Variation of the local field enhancement centered on the gap with nanorod radius and (**b**) variation of the resonance wavelength with nanorod radius.

The three design parameters, i.e., the radii of the three tips, that may be adjusted to provide high local field enhancement are one of the key advantages of the proposed tapered nanorod design. The large nanorod's radius has been tuned, and the radii of the other tips have been increased and reduced by the same factor. For the suggested design, the impacts of the nanorod radius are examined. Figure 10a depicts the intensity enhancement centered on the gap of the suggested design as a function of the radius of the large rod. The local field of the nanoantenna is enhanced to its maximum value of 45,000 at a radius of around 15 nm by tuning the radii of tapered nanorods.

The variation of the resonance wavelength as a function of the radius of the nanorod is seen in Fig. 10b. The resonance wavelength shifts toward a shorter wavelength of 6.2 µm as the radius increases from 10 to 20 nm. Furthermore, as shown in Fig. 10b, the desired wavelength of the maximum local field enhancement (6.6 µm) can be obtained at a radius of 15 nm. As a result, the radius of the nanorod can play a crucial role in controlling the resonant wavelength of the electromagnetic radiation of the suggested nanoantenna design.

**Figure 11:**
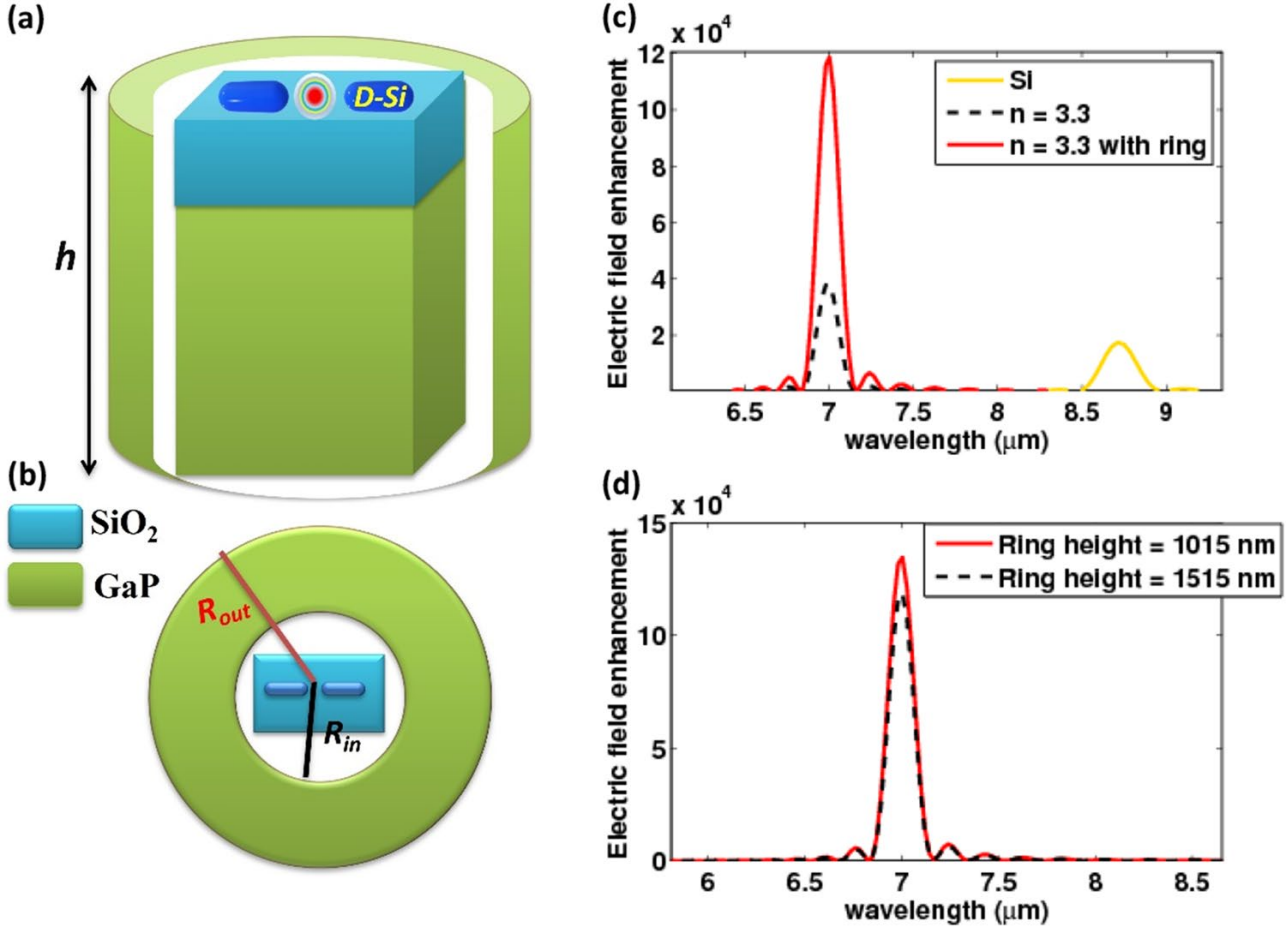
(**a**) cross sectional view of the nanorod antenna with a ring, (**b**) a top view of the nanorod antenna with a ring (**c**) wavelength-dependent local field enhancement of the hybrid nanoantenna compared to the hybrid nanoantenna with a ring and the nanoantenna with a silicon substrate, and (**d**) local field enhancement as a function of wavelength for different ring heights.

Now consider the coupled system depicted in Fig. 11a,b. The coupled system is made up of a ring that surrounds the previously mentioned nanorod antenna and the dielectric substrate. The surrounding medium index is also one. The coordinate system's origin is set in the middle of the dielectric substrate. A plane wave also excites the structure. A dielectric rectangular-ring structure has been reported to have excellent magnetic and electric field enhancements around it^17,32^. As a result, it might be a promising approach for further optimizing the electric near-field of a highly doped silicon nanoantenna. A schematic of such a coupled structure with typical incident radiation is shown in Fig. 11. The incident wave is polarized along the *z*-axis. The dielectric rectangular has a width and height of 1750 and 980 nm, respectively. The ring has 1015 nm in height with an inner and outer radius of 2500 and 5000 nm, respectively. Both the substrate and the ring have refractive indices of *n* = 3.3. a spacer is placed between the doped silicon nanorod antenna and the dielectric substrate. The spacer has a height of 5 nm and a refractive index of n = 1.46. The geometrical parameters of the doped silicon antenna are the same as in the dielectric coupled structure, which is analogous to the structure without a ring Fig. 7c. The local electric field is significantly enhanced in the gap center of the doped silicon nanoantenna. This is due to the combination of dielectric structure field enhancement (coupled structure) and doped silicon antenna field enhancement. The electric field enhancement inside the center of the gap increases to 134,779 when the height of the ring is extended to 1515 nm, while the resonant wavelength remains constant as shown in Fig. 4a. The nanorod antenna parameters used produce a sharp resonance peak of about = 7.0042 µm in Fig. 11d.

**Figure 12:**
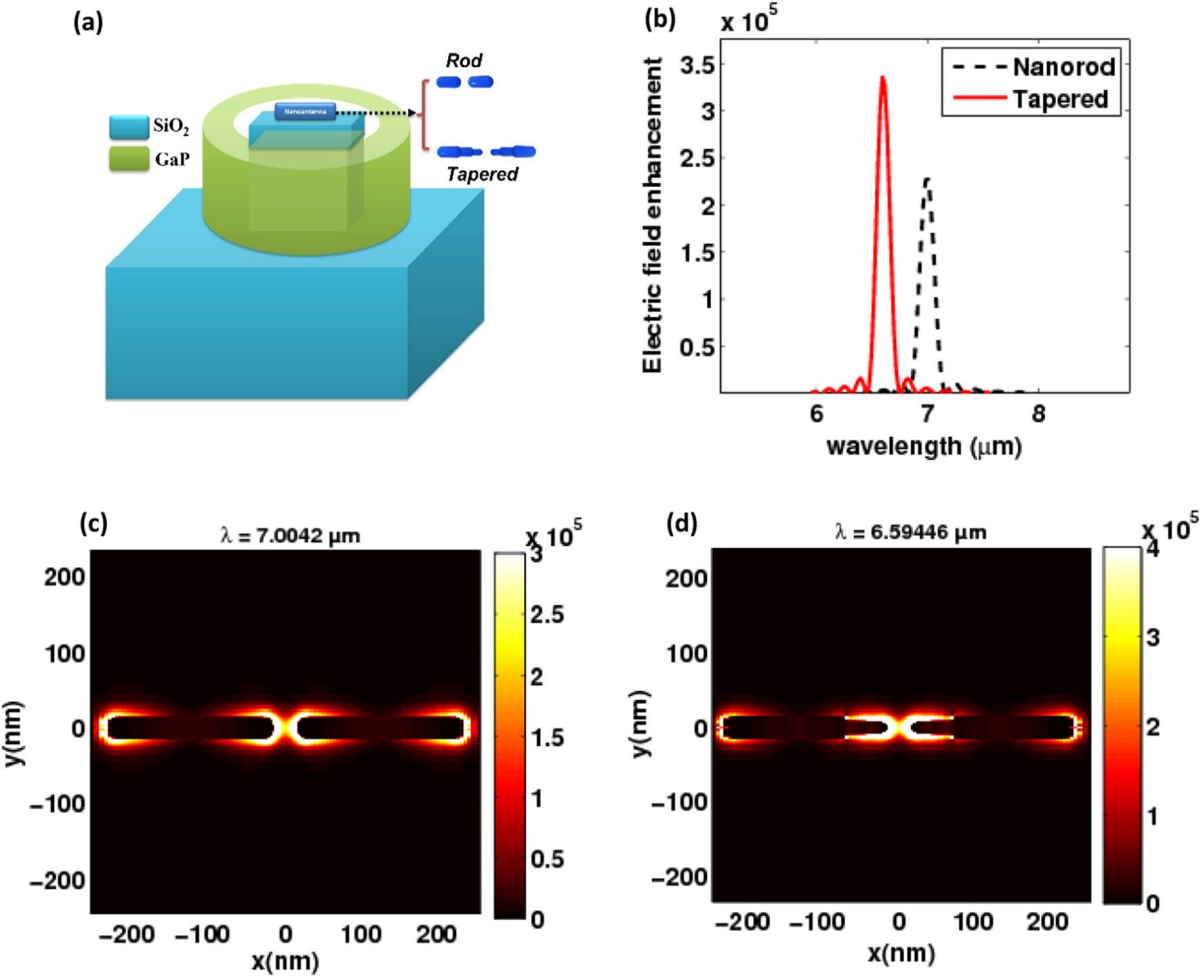
(**a**) The nanorod and tapered coupled structure on a substrate of n = 1.46, (**b**) the electric field enhancement inside the gap as a function of the wavelength, and (**c**) the corresponding electric field profiles at the resonance wavelength.

In particular, we address certain experimentally possible situations in which the nanoantenna dielectric coupled structure is situated on a substrate. The schematic of such systems is shown in Fig. 12a. SiO_2_ with a refractive index of 1.46 is assumed to be the substrate. The nanoantenna dielectric coupled structure is similar to that seen in Figs. 7 and 9. Figure 12c depicts the electric field enhancement at the center of the gap for the coupled doped silicon nanorod antenna and the coupled tapered nanoantenna. The resonant intensity enhancement in the coupled structure gives a high value, similar to what occurs in the absence of a substrate. In comparison to the case without the substrate Figs. 7 and 9, the resonance wavelength remains constant. The electric near-field profiles are estimated as well, as shown in Fig. 12b, and they are comparable to the case without the substrate.

**Figure 13:**
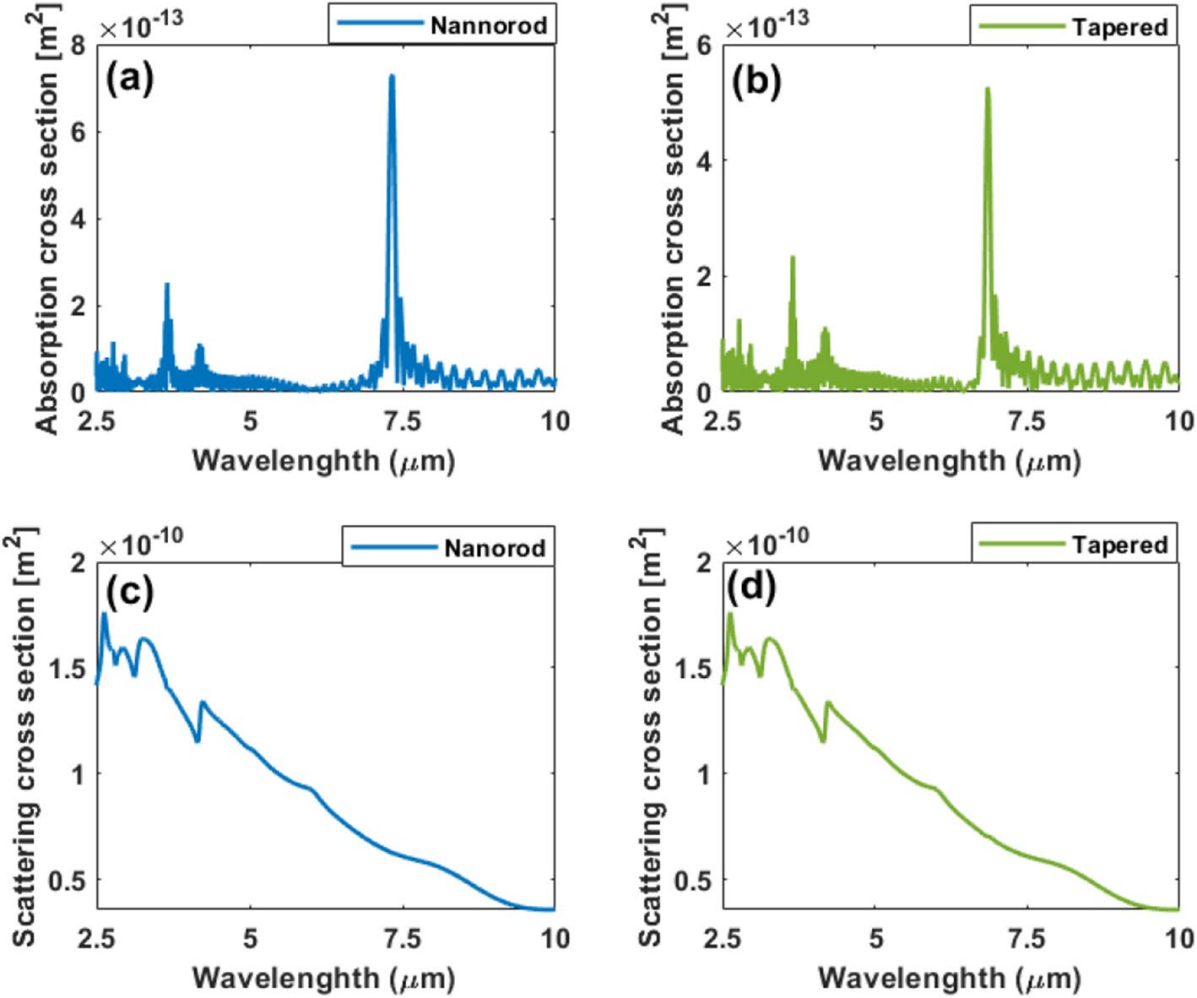
(**a**,**c**) the cross-section spectra of the nanorod with the coupled structure on a substrate. (**b**,**d**) the cross-section spectra of the tapered nanoantenna with the coupled structure on a substrate.

On their scattering spectrum (Fig. 13a,b), the nanorod coupled system has a sharp resonance peak at λ = 7.0042 µm, whereas the tapered coupled structure has the main peak at *λ* = 6.59446 µm, which is dominated by a magnetic dipole resonance. Several previous studies have found similar results^17,32,33^. This strong far-field resonance frequently results in a significant electric field enhancement around the coupled structure, as seen in Fig. 12. The plasmonic-based doped silicon nanorod antenna has hybridized mode of two electric dipole plasmon resonances of the rods is approximate at *λ* = 7.0042 µm and the resonance of the tapered nanorod antenna is at *λ* = 6.59446 µm which is about the magnetic dipole mode of the coupled structure. The same results have also been achieved in the literature^17,32^. Figure 13c,d show the scattering cross section for the nanorod antenna and the tapered nanoantenna, respectively.

**Figure 14:**
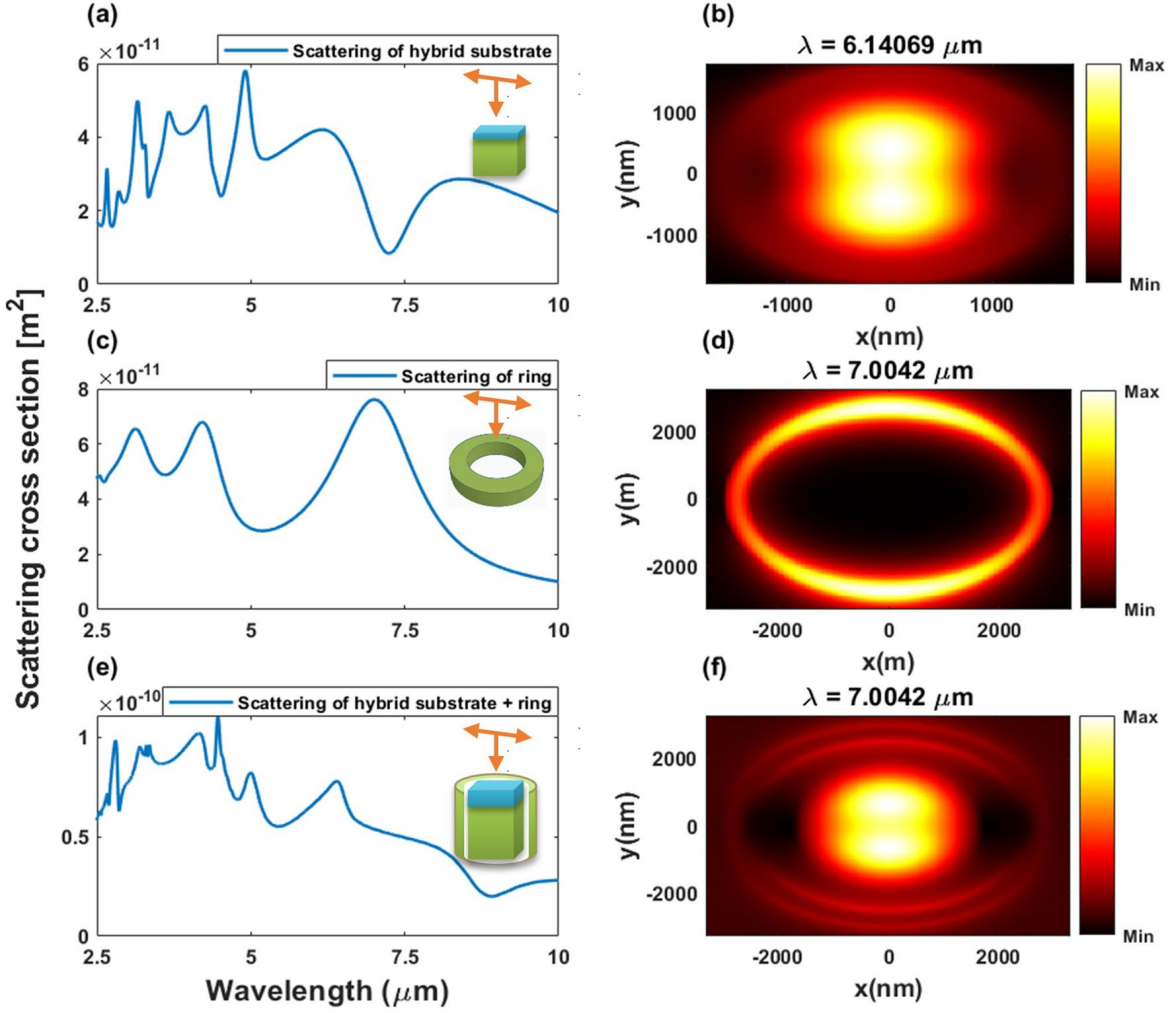
Optical responses of the hyrid substrate, ring, and the coupled system. (**a**,**c**,**e**) are the scatterin spectrums of the individual hybrid substarte, the individual ring, and the coupled system. The schematic of the structure with TFSF excitation is shown in the bottom inset. (**b**,**d**,**f**) are the corresponding magnetic field distribution on the x–y plane.

Figure 14 exhibits the scattering cross-section spectrum of the individual hybrid substrate, individual ring, and the coupled system (hybrid substrate with ring). A normal incident TFSF excites the hybrid substrate. The heights of the substrate (*n* = 3.3) and spacer (*n* = 1.46) are about *l*_*t*_ = 980 nm and *l*_*s*_ = 5 nm, respectively, and both widths are *w* = 2000 nm. The origin of the coordinate system is located at the center of the hybrid substrate. The scattering cross-section of the individual substrate (Fig. 14a) shows a resonance peak at *λ* = 6.14069 μm. This peak is associated with a magnetic dipole mode, as indicated by the magnetic field enhancement (|*H*|^2^/|*H*_0_|^2^) distribution in Fig. 14b.

The ring has a height of (*h* = 1015 nm). The inner and outer radii of the ring are *R*_*in*_ = 2500 and *R*_*out*_ = 5000 nm, respectively. A resonant peak is shown about *λ* = 7.0042 μm with the specified geometrical parameters (Fig. 14c). This resonance position is close to that of the magnetic dipole mode obtained in the case of the hybrid substrate. In order to figure out this mode, the magnetic near field distributions in the *y*-direction (|*H*_*y*_|/|*H*_0_|) are also simulated (Fig. 14d).

Now, the coupled system in Fig. 14e is also investigated. The coupled system is composed of the hybrid substrate and ring mentioned above. The surrounding medium index is 1. The origin of the system’s coordinate is located at the center of the hybrid substrate (Fig. 14e). A TFSF with normal incidence excites the structure. The scattering cross-section in Fig. 14e shows the coupling between the hybrid substrate and the ring. A resonant peak can be shown at *λ* = 6.14069 μm. This peak value is close to the individual substrate and ring resonance positions. In order to indicate the magnetic dipole mode at the resonance wavelength of the nanorod antenna, the magnetic field distribution (|*H*|^2^/|*H*_0_|^2^) has also been computed at *λ* = 7.0042 μm. It can be shown that the magnetic distribution also identifies the magnetic dipole mode for the coupled system as shown in Fig. 14f.

**Figure 15:**
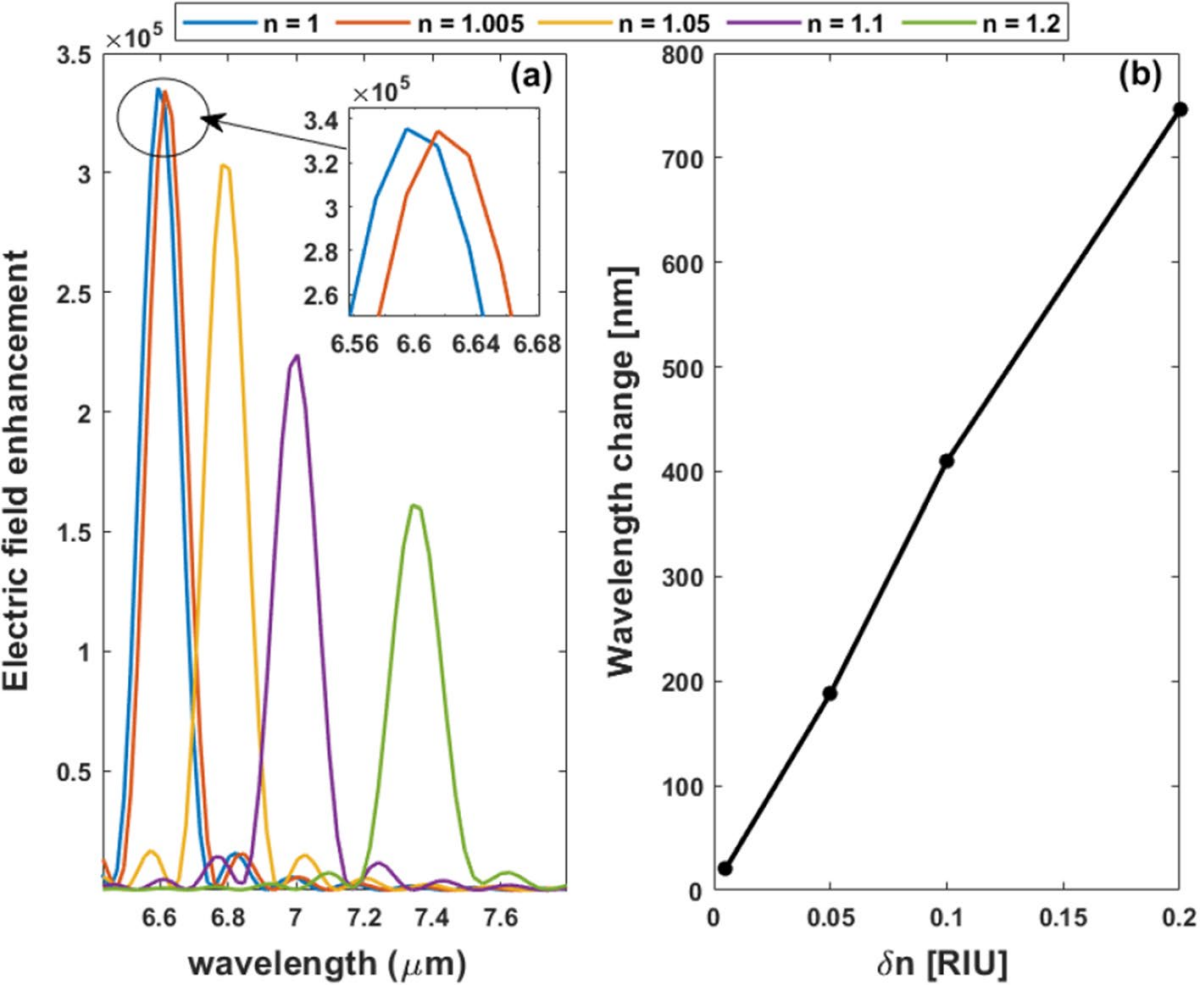
Electric field intensity at the center of the gap (*x* = 0, *y* = 0) as a function of the wavelength for different medium refractive indices and (**b**) the resonance wavelength shift with varying RI for the tapered nanoantenna on the coupled structure with a substrate.

The effect of changing the RI of the surrounding on the intensity of the tapered nanoantenna shown in Fig. 12a has been also investigated by using FDTD simulations. The difference between air and gas field intensities is seen in Fig. 15a which reveals a significant change in the resonance peaks between air and different medium indices. The tapered design exhibits a red shift in the resonant wavelength, however, the field enhancement is decreased, as illustrated in Fig. 15a. Furthermore, the sensitivity of the medium refractive index change is studied as shown in Fig. 15b. The maximum sensitivity reaches 3729 nm/RIU.

## Conclusion

In conclusion, two different designs for the doped silicon dipole nanoantennas are proposed and studied using the Lumerical software package based on the 3D FDTD approach. The suggested dielectric nanoantenna enhances the local field intensity at the MIR. The goal of the study is to develop high-doped silicon-based nanoantennas that exhibit Localized Surface Plasmon Resonance (LSPR), comparable to plasmonic metal nanoantennas. The proposed nanoantenna is made up of two highly doped nanorod structures that are stacked on top of a dielectric substrate. It is found that the doped silicon nanoantenna exhibits strong local field enhancement at MIR wavelengths. Furthermore, the suggested design has lower dissipation losses.

When compared to the nanoantenna in a silicon substrate, the hybrid substrate composition exhibits a significant enhancement; the enhancement value is around two-fold. The geometry of the nanoantenna influences the peak of the field enhancement and its resonance, with the nanorod and tapered nanoantennas achieving a high level of field enhancement. The coupled system with the incorporation of the ring around the nanorod leads to a condition in an ultrahigh enhancement field while remaining the same resonance wavelength. This enhancement is attributed to the integration of nanoantenna which has hybridized mode of two electric dipole plasmon resonances due to the two nanorods with a gap of 30 nm which is roughly the magnetic dipole mode of the dielectric substrate.

## Data Availability

The datasets used and/or analyzed during the current study are available from the corresponding author on reasonable request.
